# A Preliminary Study on Infrared Thermograph of Metabolic Syndrome

**DOI:** 10.3389/fendo.2022.851369

**Published:** 2022-04-12

**Authors:** Meng-jiao Gao, Hui-zhong Xue, Rui Cai, Bi-yao Jiang, Bao-hong Mi, Zong-jun Chen, Yin-chun Shi, Yong-hua Xiao, Wen-zheng Zhang

**Affiliations:** ^1^ The First Clinical Medical School, Beijing University of Chinese Medicine, Beijing, China; ^2^ Beijing Wholelife Medical Science Co., Ltd., Beijing, China; ^3^ School of Acupuncture-Moxibustion and Tuina, Beijing University of Chinese Medicine, Beijing, China; ^4^ Dongzhimen Hospital, Beijing University of Chinese Medicine, Beijing, China

**Keywords:** metabolic syndrome, infrared thermography, temperature, evaluation method, hypertension, hyperglycemia, abdominal obesity

## Abstract

**Objective:**

To explore the temperature distribution characteristics of the face, palms, feet and the trunk area of metabolic syndrome (MS) through infrared thermography (IRT) and provide evidence for the application of IRT in the assistant evaluation of MS population.

**Methods:**

We collected thermographs of 184 participants (91 males, 93 females) and further divided participants of each gender into 4 groups according to the number of abnormal metabolic indexes. Mean temperatures of 6 Region of Interests (ROIs) (face, anterior trunk, bilateral palms and dorsum of feet) were calculated. Comparisons of the mean temperatures between genders, among groups and ROIs were carried out.

**Results:**

Male participants had higher mean temperature in their face, palms (*P*<0.01) and dorsum of feet (*P*<0.05), and lower mean temperature in the anterior trunk (*P*<0.01). Female participants with MS had higher mean temperature in their palms and dorsum of feet (*P*<0.01) and lower mean temperature in the anterior trunk (P<0.01) than normal participants. Similar tendencies were shown in the mean temperature of the left palms and trunk of MS males. With the increase of the number of abnormal metabolic indexes, it seems that the mean temperature gradually increased in palms and dorsum of feet, and decreased in the anterior trunk.

**Conclusion:**

The thermograph of MS exhibits certain characteristics. This may help reveal the correlations between Infrared thermography and metabolic disorders.

## 1 Introduction

Metabolic Syndrome (MS) is a clinical syndrome characterized by the presence of a group of metabolic disorders, including hyperglycemia (Diabetes Mellitus, DM, or Impaired Glucose Regulation, IGR), dyslipidemia (hypertriglyceridemia and/or low high density lipoprotein cholesterol), hypertension and central obesity (1). Evidence indicates that MS significantly promotes the onset and progression of type 2 diabetes mellitus (T2DM), cardiovascular and cerebrovascular diseases. Compared with patients without MS, MS patients had a higher risk and mortality of cardiovascular disease (CVD) (with a relative risk of 2.35 and 2.40, respectively) (2). In 2013-2014, the prevalence of MS adults was 31.5% in the US (3). In Mainland China, the prevalence of MS among the population aged 15 years and older has reached 24.5% in 2016 (4). According to the Yearbook issued by China’s National Health and Family Planning Commission, the total cost of hospitalization for diabetes, acute myocardial infarction and cerebral infarction in China has reached RMB 49.1 billion in 2016 (5), more than three times higher than that in 2012 (6). The increasing prevalence of MS implicates a heavy global health burden and socioeconomic cost, making it of great significance for early diagnosis and intervention of MS.

Nevertheless, in China, 2015, the awareness rate, treatment rate, and control rate of hypertension among adults were 51.6%, 45.8%, and 16.8%, respectively (7). In the 2013 national survey, undiagnosed diabetics accounted for 63% of the total (8). In 2010, the awareness rate, treatment rate, and control rate of dyslipidemia in Chinese adults were 10.93%, 6.84%, and 3.53%, respectively (9). The unsatisfying control station of MS might be the results due to asymptomatic MS patients of early-stage and complicated diagnostic procedure, which involves multiple assessments of body indexes such as blood glucose, blood lipid, blood pressure, waist circumference, etc., some of which are invasive examination and inconvenient to patients. Hence, it’s meaningful to explore a method that can effectively and quickly pick out MS patients as well as the high-risk groups of MS from so-called “healthy people”, assisting early detections and evaluation of the MS population.

Infrared thermography (IRT) is a technique capable of capturing infrared radiation emanating from the human body and converting it to temperature with the output of thermal maps (10). This accurate (with a thermal resolution of 0.03-0.1°C), non-contact and non-invasive technique has been used to early screening and efficacy evaluation of numerous diseases such as breast cancer, diabetic neuropathy, liver metastases, cardiovascular disease and so on (11, 12). The body surface temperature is mainly affected by local blood perfusion, but the influence of muscle activity and metabolic activity cannot be excluded (13). It might serve as an indicator of the function of corresponding parts of the body. Therefore, it’s possible to detect diseases before the noticeable occurrence of structural changes.

According to recent studies, the metabolic indexes which are used in the diagnosis of MS do have correlations with skin temperature: temperature of palms of DM patients tend to be lower than that of normal people (14). While overweight females have lower mean abdominal temperature and higher hand temperature than the lean ones (15), and average skin surface temperature (°C) waveform of hypertension participants varies from normal participants (16). Besides, skin temperature of the anterior supraclavicular shows the ability to detect brown adipose tissue (BAT) activation, which is involved in the body weight, glucose and lipid regulation, reflecting the metabolic changes (17, 18). Therefore, IRT exhibits the potential of indicating metabolic disorders, and may have some advantages and application prospects in MS screening.

Based on the current findings, we conducted this study which sought to explore the temperature distribution characteristics of the face, trunk, palms and feet of MS, hope to establish the correlation between specific thermograph patterns and metabolic disorder, and provide reference to further studies and the application of IRT in early assistant diagnosis and evaluation of MS population.

## 2 Materials and Methods

### 2.1 Participants

The data of this retrospective study were collected from patients who visited the Health Management Center of the International Department of Dongzhimen Hospital and the endocrinology department of Dongzhimen Hospital, Beijing University of Chinese Medicine from June 2016 to April 2019. After reviewing the inclusion, exclusion and rejection criteria, 184 Asian participants aged between 18 and 70 were brought into the study, including 91 males and 93 females. This study has been waived the requirement for informed consent by the institutional Ethics Committee of Dongzhimen Hospital because of its retrospective nature (No. DZMEC-KY-2020-12).

The MS diagnostic criteria used in the study was published in *Guidelines for the prevention and control of type 2 diabetes in China (2017 Edition)* (19) by Chinese Diabetes Society: 1) Abdominal obesity: waist circumference ≥ 90 cm in males and 85 cm in females; 2) Hyperglycemia: fasting plasma glucose ≥6.1 mmol/L or 2-hour plasma glucose ≥ 7.8mmol/L following a 75 g oral glucose load and (or) diabetes diagnosis has been confirmed; 3) Hypertension: blood pressure≥130/85 mmHg and (or) hypertension diagnosis has been confirmed; 4) Fasting TG≥1.70 mmol/L; 5) Fasting HDL-C<1.04 mmol/L; 3 out of 5 factors above are required.

Exclusion criteria: 1) Coronary heart disease, cerebral vascular disease, hypertensive nephropathy, diabetic retinopathy, diabetic peripheral neuropathy, diabetic peripheral vascular disease, diabetic nephropathy or other severe chronic complications caused by MS constituents (hypertension, hyperlipidemia, diabetes); 2) Menstruation, pregnancy or lactation; 3) Diseases that may affect body temperatures, such as cold and thyroid disease; 4) Medical histories of hepatic and renal insufficiency, hematopathy, tumor, severe trauma or major surgery; 5) Critical illness such as diabetic ketoacidosis, hypertensive crisis and hypertensive encephalopathy.

Rejection criteria: 1) Data with obvious error, low creditability or omissions; 2) Thermal maps taken in non-standard positions, blurring or interfered by sweat or medical ultrasonic couplant.

### 2.2 Groups

The participants were grouped by their gender (group M for males and group F for females) and further divided into four groups according to the number of abnormal metabolic indexes.

1) Two normal groups (M0, F0) with no abnormal metabolic index, consist of 26 participants in group M0 and 50 participants in group F0;

2) Two groups with 1 abnormal index (M1, F1), consist of 18 participants in group M1 and 21 participants in group F1;

3) Two groups with 2 abnormal indexes (M2, F2), consist of 16 participants in group M2 and 10 participants in group F2;

4) Two MS groups (M3, F3) with 3 or more abnormal indexes, consist of 31 participants in group M3 and 12 participants in group F3.

### 2.3 Methods

The following data of all participants were collected: demographics (gender, age), medical history, body measurement indexes (height, body mass, BMI, waist circumference, hip circumference), systolic blood pressure (SBP) and diastolic blood pressure (DBP), fasting plasma glucose (FPG), total cholesterol (TC), triglyceride (TG), high density lipoprotein cholesterol (HDL-C), low density lipoprotein cholesterol (LDL-C), serum uric acid (UA), etc.

Thermography and all those data above of each participant were obtained on the same day. Thermography was captured by an HIR-2000A (FLIR detector) medical infrared camera produced by Beijing Yuetian Optoelectronics Technology Co., Ltd, with the emissivity of 0.98, spectral range of 8-14μm, frame rate of 9 frames/second, pixels/frame of 256×324×14 Bits, thermal resolution of 0.05°C, and spatial resolution of 0.95 mrad. The detector had gone through blackbody correction and temperature calibration before leaving the factory. The ambient temperature was controlled at 22.0°C ± 2.0°C, and the relative humidity was 60% - 70%. There were no other electronic devices, no air convection nor direct illumination of strong light in the cabin. The participants were informed to avoid taking in foods that were too cold or too hot one hour before the conduction of thermography, loosen the clothing, take off hats and glasses, avoid pressing or scratching the body, and rest for 15 minutes before the examination. The participants were unclothed and kept 2 meters away from the detector. Both anterior and posterior views were captured in the anatomical position (the gesture of standing erect with palms facing forward).

**Figure 1:**
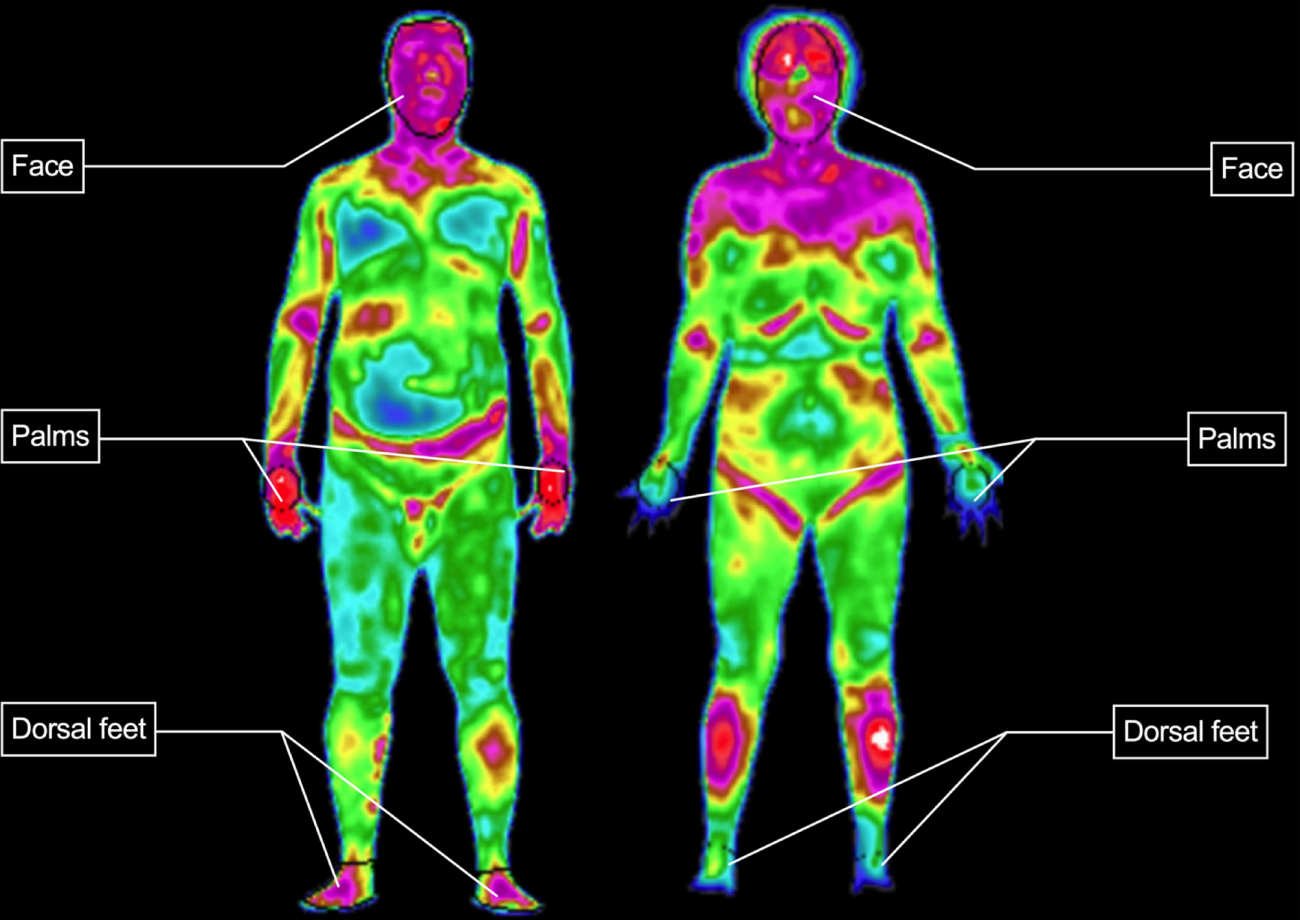
Delimitation of ROI.

The analysis of thermography was carried out by TMI-W Infrared Medical Imaging Workstation V1.0 produced by Beijing Wholelife Medical Science Co., Ltd. Mean temperature of the anterior trunk was generated automatically by the workstation. The other five regions of interest (ROIs) (face, bilateral palms and bilateral dorsum of feet) were manually delimited (**Figure 1**). Then mean temperature (T_mean_ ± SD) of each ROI were calculated (represented as T_f_ for the face, T_t_ for the anterior trunk, T_rp_/T_lp_ for the right/left palm, T_rf_/T_rf_ for the right/left dorsum of the foot).

### 2.4 Outcomes

The primary objectives of the analysis were to compare the mean temperature of six ROIs within each group to find out the temperature distribution characteristics of each group that conform to different numbers of indexes in MS diagnostic criteria, and to compare the mean temperature of four groups in each gender to explore the fluctuation trends of temperature along with the progress of metabolic disorders. And the secondary objective of the analysis was to probe into the correlation of the mean temperature of the ROIs with the measurement and laboratory data that relevant to metabolic disorders.

### 2.5 Statistical Analysis

**Figure 2:**
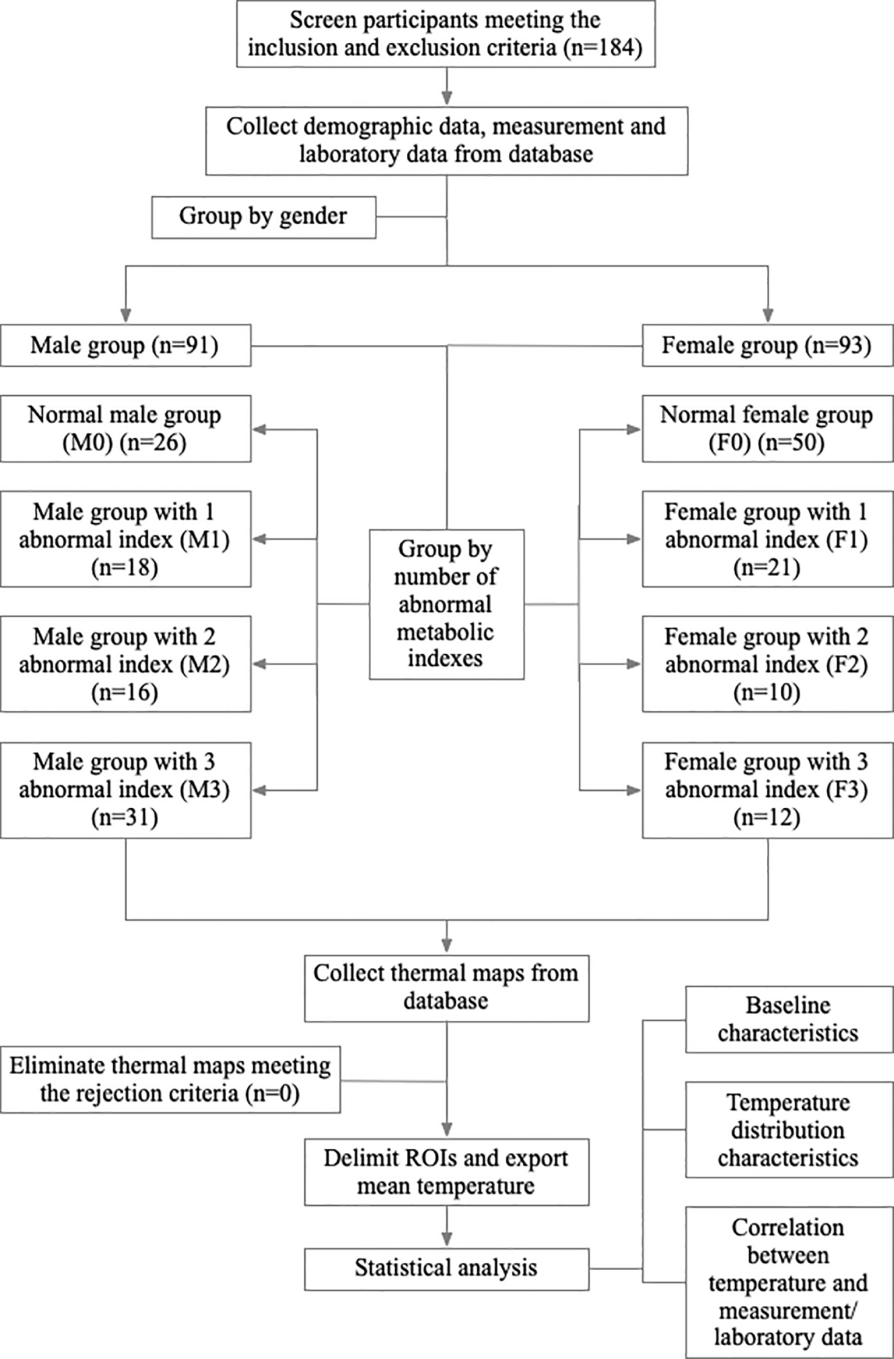
Study flow chart.

Statistical analysis was carried out using IBM^®^SPSS^®^Statistics (version 24). Data were tested for normality using the Shapiro –Wilk test (α=0.1). Variable transformation was performed for data not conforming to normal distribution. With regard to the quantitative data with normal distribution, differences between two groups were tested using the two-sample t-test for data with homogeneity and t’-test for data with heterogeneity of variance. One-way analysis of variance (ANOVA) followed by Bonferroni’s *post-hoc* test was used to compare multiple groups. For the data which did not conform to the normal distribution or homogeneity of variance, differences between two groups or multiple groups were tested using Wilcoxon rank-sum test and Kruskal-Wallis test, respectively. The measurement data were expressed as mean ± standard deviation $\left(\bar{\text{x}}\pm \text{SD}\right)$ . Correlation analysis was performed using the Pearson correlation test. Then perform unary linear regression or multiple stepwise regression to analyze the correlations between T_mean_ of ROI and correlated measurement and laboratory data. In regression analysis, T_mean_ of each ROI was set as the dependent variable, and independent variables were corresponding data. For all statistical analyses, a p-value lower than 0.05 was assumed significant. The complete study process is shown in **Figure 2**.

## 3 Results

### 3.1 Baseline Characteristics

Demographic and clinical data are summarized in **Table 1**. The age distribution was similar in male groups while having significant differences (P<0.05) in female groups. With the increase of the number of abnormal metabolic indexes, except the age of women increased significantly, some of the body measurement and laboratory data also showed increasing trend among four subgroups in both genders (**Table 1**).

**Table 1:** Baselines of age, measurement and laboratory data in groups (mean, minimum-maximum).

Groups	M0	M1	M2	M3	χ^2^/F^4^	*P*	F0	F1	F2	F3	χ^2^/F^4^	*P*
n	26	18	16	31			50	21	10	12		
Age (years)	36.46 (23-58)	36.60 (25-63)	41.00 (24-61)	43.16 (28-66)	2.788^1^	0.045	33.50 (21-55)	41.76 (27-60)	44.10 (28-61)	55.58 (38-69)	16.027^1^	0.000
Height (cm)	172.55 (165.5-181.8)	174.13 (167.0-185.4)	173.24 (160.0-184.1)	173.64 (155.0-187.0)	0.305^2^	0.822	161.82 (150.5-177.8)	161.88 (147.7-170.3)	163.40 (155.8-171.0)	160.07 (155.2-166.4)	2.708^3^	0.439
Body mass (kg)	68.20 (60.6-79.75)	71.18 (59.5-82.1)	79.03 (64.0-99)	88.31 (65.0-110.0)	46.586^3^	0.000**	54.74 (43.6-72.6)	57.79 (43.8-74.6)	66.84 (56.1-78.8)	64.62 (52.0-82.2)	19.548^3^	0.000**
BMI (kg/m^2^)	22.85 (20.84-27.15)	23.49 (21.04-25.89)	26.28 (21.94-32.18)	29.31 (23.34-38.51)	51.894^3^	0.000**	20.90 (16.52-26.63)	22.03 (15.97-25.90)	25.10 (20.52-31.39)	25.30 (20.06-33.78)	20.243^3^	0.000**
Waist circum-ference (cm)	81.24 (72.0-87.0)	86.22 (73.0-93.0)	93.06 (78.0-111.0)	100.20 (88.0-124.0)	58.219^3^	0.000**	72.11 (60.0-84.0)	77.50 (68.0-91.0)	82.40 (70.0-101.0)	85.75 (72.0-99.0)	30.965^3^	0.000**
Hip circum-ference (cm)	94.87 (87.0-103.0)	97.26 (92.0-103.0)	101.68 (92.0-112.0)	103.73 (93.3-116.0)	37.062^3^	0.000**	91.69 (81.0-104.0)	94.13 (84.0-107.0)	98.90 (91.0-110.0)	95.00 (87.0-105.0)	5.700^2^	0.001**
SBP (mmHg)	115.89 (101-129)	119.78 (107-137)	123.88 (100-151)	134.84 (99-172)	19.902^3^	0.000**	110.00 (97-127)	119.05 (94-145)	126.80 (102-151)	124.08 (107-140)	17.729^3^	0.001**
DBP (mmHg)	68.85 (61-78)	72.00 (60-87)	77.56 (59-97)	85.48 (53-112)	27.852^3^	0.000**	66.86 (53-85)	73.19 (57-96)	78.40 (65-94)	77.25 (55-110)	7.743^2^	0.000**
FPG (mmol/L)	5.09 (4.38-5.74)	5.57 (4.58-10.74)	5.73 (4.37-8.54)	7.27 (4.53-17.06)	21.156^3^	0.000**	4.96 (4.28-5.86)	5.14 (4.34-7.11)	5.56 (5.07-6.23)	7.58 (4.94-11.19)	30.419^3^	0.000**
UA (μmol/L)	352.0 (328.1-375.9)	364.7 (281.5-485.9)	402.8 (309.5-488.6)	414.7 (271.4-552.5)	4.831^2^	0.004**	253.9 (119.4-459.1)	252.3 (137.7-414.5)	289.8 (231.1-365.5)	306.1 (206.7-434.33)	10.683^3^	0.014*
TC (mmol/L)	4.95 (3.71-6.39)	4.98 (3.82-7.57)	5.06 (3.56-7.00)	5.08 (3.16-8.84)	0.719^3^	0.869	4.87 (3.22-7.62)	4.83 (3.52-6.01)	5.44 (3.15-7.02)	5.18 (4.20-7.25)	6.483^3^	0.090
TG (mmol/L)	0.91 (0.32-1.69)	1.25 (0.65-2.58)	1.65 (0.73-4.17)	2.88 (0.71-9.64)	46.253^3^	0.000**	0.78 (0.27-1.33)	1.22 (0.46-2.59)	1.40 (0.84-2.23)	2.20 (0.80-4.83)	33.767^3^	0.000**
HDL-C (mmol/L)	1.41 (1.07-2.57)	1.29 (0.88-3.14)	1.18 (0.71-1.85)	1.07 (0.63-2.18)	23.975^3^	0.000**	1.57 (1.16-2.54)	1.45 (0.95-1.90)	1.38 (0.79-2.40)	1.17 (0.84-1.52)	18.806^3^	0.000**
LDL-C (mmol/L)	2.79 (1.84-4.07)	2.91 (1.13-5.00)	3.02 (1.47-4.24)	2.82 (0.78-5.61)	0.372^2^	0.773	2.60 (1.40-4.75)	2.56 (1.81-3.87)	3.12 (1.82-4.31)	3.17 (0.79-4.84)	8.063^3^	0.045*

^1^ANOVA performed on the age of both gender groups (with variable transformation), Post-Hoc tests (Bonferroni method) showed no significance in ages between four male groups (P > 0.05), female group F1, F2 (P = 1.000), and F2, F3 (P = 0.106).

^2^For data that conformed to the normal distribution and homogeneity of variance, ANOVA was implemented.

^3^For data that did not conform to the homogeneity of variance, Kruskal-Wallis test was used.

^4^χ^2^/F refers to the χ^2^ value in Kruskal-Wallis test/F value in ANOVA.

^*^P < 0.05, ** P < 0.01.

### 3.2 Characteristics of T_mean_ of 6 ROI in Different Gender Groups

**Figure 3:**
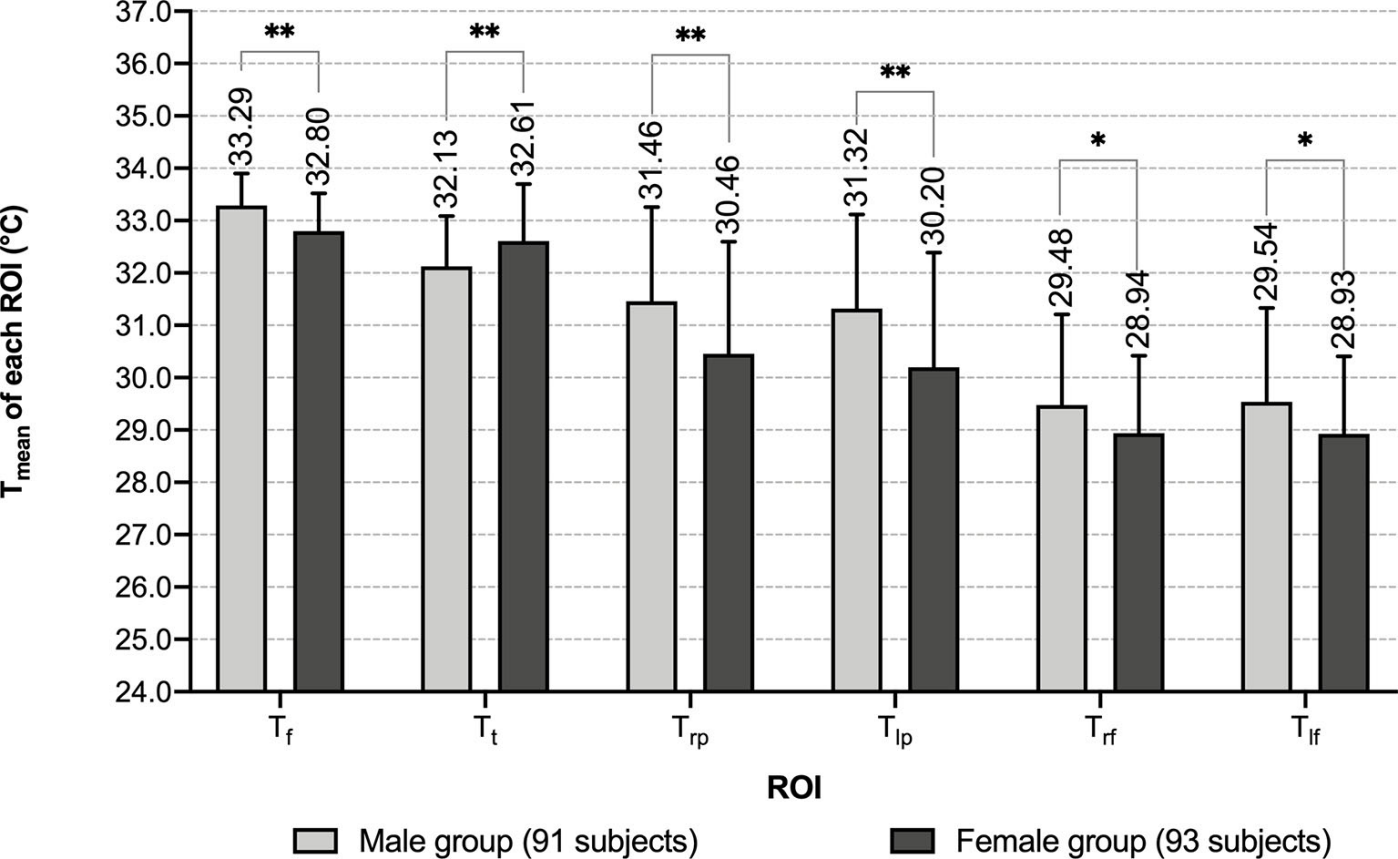
Comparison of T_mean_ of each ROI between different genders. *: *P* < 0.05 **: *P* < 0.01.

Compared with females, males had higher mean temperatures in the areas of face, palms and dorsum of feet, and lower mean temperature in anterior trunk (*P*<0.05). These diversities indicated that gender difference significantly influences T_mean_ of each ROI. Therefore, it is necessary to discuss characteristics of each ROI grouping by gender (**Figure 3** and **Supplementary Table 1**).

#### 3.2.1 Characteristics of T_mean_ of ROI in Male Groups

**Figure 4:**
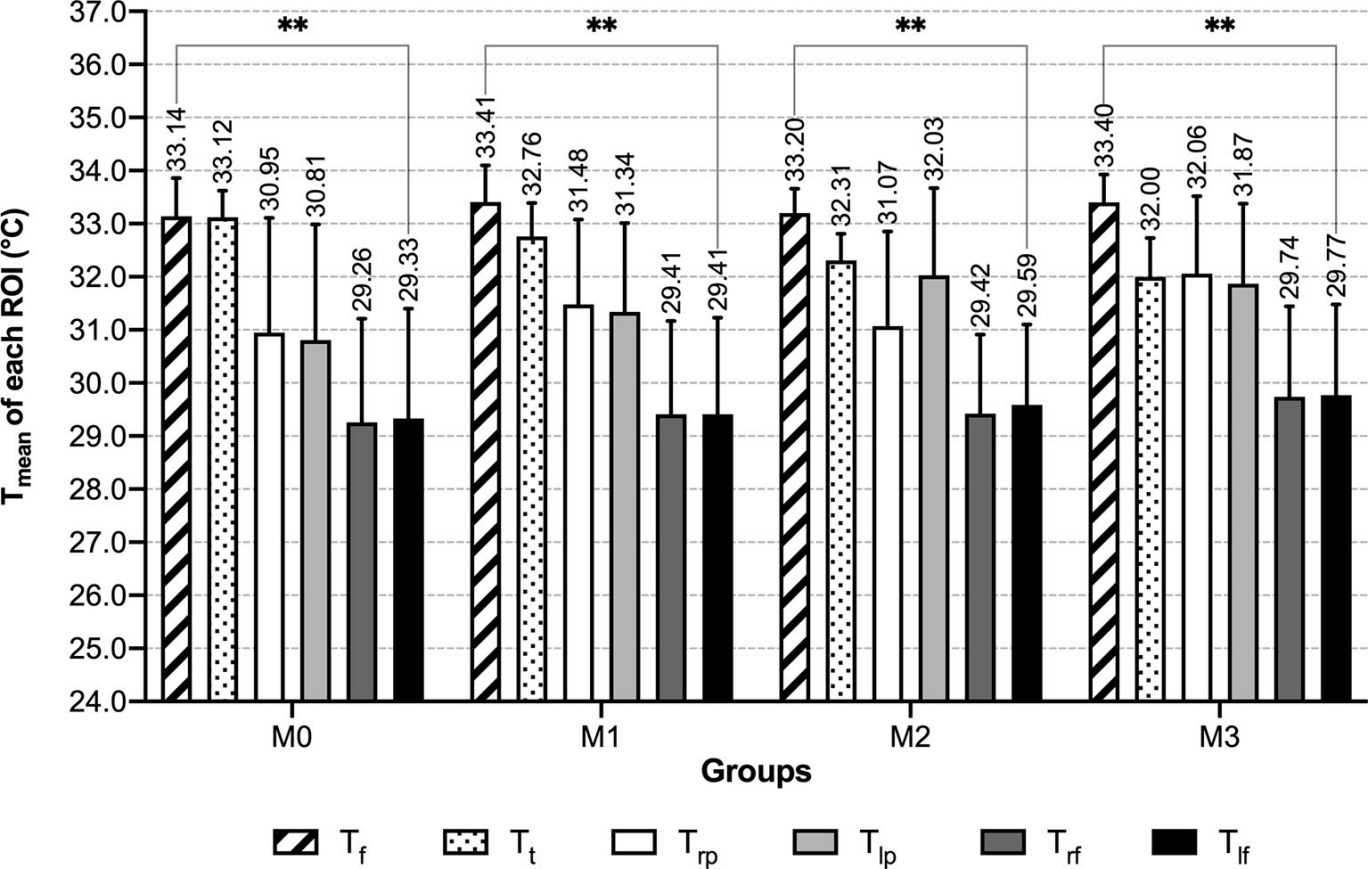
T_mean_ of each ROI of male groups - grouped by number of abnormal indexes. **: P < 0.01.

**Figure 5:**
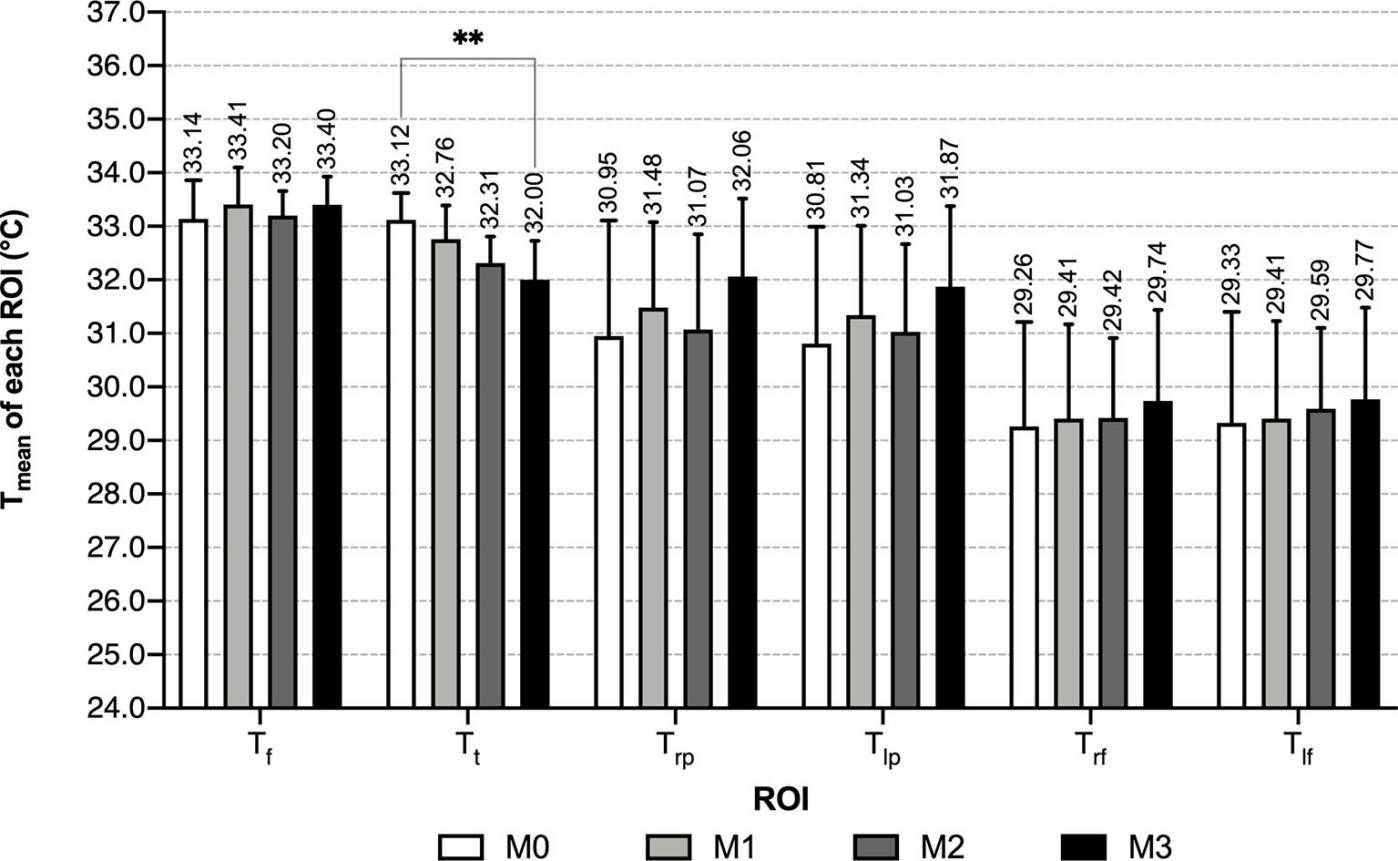
T_mean_ of each ROI of male groups - grouped by ROI. **: *P* < 0.01.

Within each male group, mean temperature exhibited a decreasing trend in the order of the face, anterior trunk, bilateral palms, bilateral dorsum of feet, and the differences among these temperature values were significant (P<0.01) (**Table 2** and **Figure 4**). With the increase of the number of abnormal metabolic indexes, Tt decreased successively from group M0 to M3. In T_mean_ of other ROIs, slightly increasing trends with no significant difference in the order of M0, M1 & M2, M3 can be recognized (**Table 2** and **Figure 5**). When focusing on the differences between group M0 and M3, MS males had higher mean temperature in their left palms and lower mean temperature in their anterior trunks (P<0.05) (**Supplementary Table 2**).

**Table 2:** Comparison of T_mean_ of each ROI among four groups in each gender $\left(\text{C};\;\bar{\text{x}}\pm \text{s}\right)$ .

Groups	M0	M1	M2	M3	χ^2^/F^4^	*P*	F0	F1	F2	F3	χ^2^/F^4^	*P*
n	26	18	16	31			50	21	10	12		
**T_f_**	33.14 ± 0.72	33.41 ± 0.69	33.20 ± 0.46	33.40 ± 0.53	1.157^2^	0.331	32.76 ± 0.84	32.88 ± 0.71	32.73 ± 0.47	32.93 ± 0.36	1.851^3^	0.604
**T_t_**	33.12 ± 0.50	32.76 ± 0.63	32.31 ± 0.50	32.00 ± 0.73	17.405^2^	0.000**	33.14 ± 0.67	32.74 ± 0.69	32.56 ± 0.88	32.33 ± 0.79	13.426^3^	0.004**
**T_rp_**	30.95 ± 2.16	31.48 ± 1.60	31.07 ± 1.78	32.06 ± 1.46	5.155^3^	0.161	29.88 ± 1.98	30.59 ± 1.93	30.64 ± 2.60	32.49 ± 1.56	14.628^3^	0.002**
**T_lp_**	30.81 ± 2.18	31.34 ± 1.67	31.03 ± 1.64	31.87 ± 1.51	5.430^3^	0.143	29.61 ± 1.94	30.19 ± 2.04	30.37 ± 2.71	32.54 ± 1.53	17.660^3^	0.001**
**T_rf_**	29.26 ± 1.95	29.41 ± 1.76	29.42 ± 1.49	29.74 ± 1.70	2.330^3^	0.507	28.43 ± 1.32	29.02 ± 0.87	28.95 ± 1.76	30.89 ± 1.14	23.742^3^	0.000**
**T_lf_**	29.33 ± 2.07	29.41 ± 1.82	29.59 ± 1.51	29.77 ± 1.71	2.128^3^	0.546	28.39 ± 1.28	29.18 ± 0.95	28.84 ± 1.87	30.82 ± 1.10	23.378^3^	0.000**
**χ^2^**	82.062^1^	60.080^1^	56.496^1^	97.698^1^			200.326^1^	67.455^1^	23.618^1^	29.322^1^		
***P* ^1^**	0.000**	0.000**	0.000**	0.000**			0.000**	0.000**	0.000**	0.000**		

^1^Kruskal-Wallis test was used for comparing T_mean_ of six ROI within each gender group.

^2^ANOVA followed by Bonferroni’s post-hoc test was implemented for comparing T_mean_ of each ROI between four male groups. In post-hoc test, significance was found between T_t_ of group M0/M2, M0/M3, M1/M3 (adjusted P<0.05).

^3^When comparing T_mean_ of each ROI between four groups in each gender, for data that did not conform to the normal distribution, Kruskal-Wallis test was used. Bonferroni correction showed significance between T_t_ and T_rp_ of group F0/F3, T_lp_ of group F0/F3, F1/F3, T_rf_ and T_lf_ of group F0/F3, F1/F3, F2/F3 (adjusted P < 0.05).

^4^χ^2^/F refers to the χ^2^ value in Kruskal-Wallis test/F value in ANOVA. ** P < 0.01.

#### 3.2.2 Characteristics of T_mean_ of 6 ROI in Female Groups

**Figure 6:**
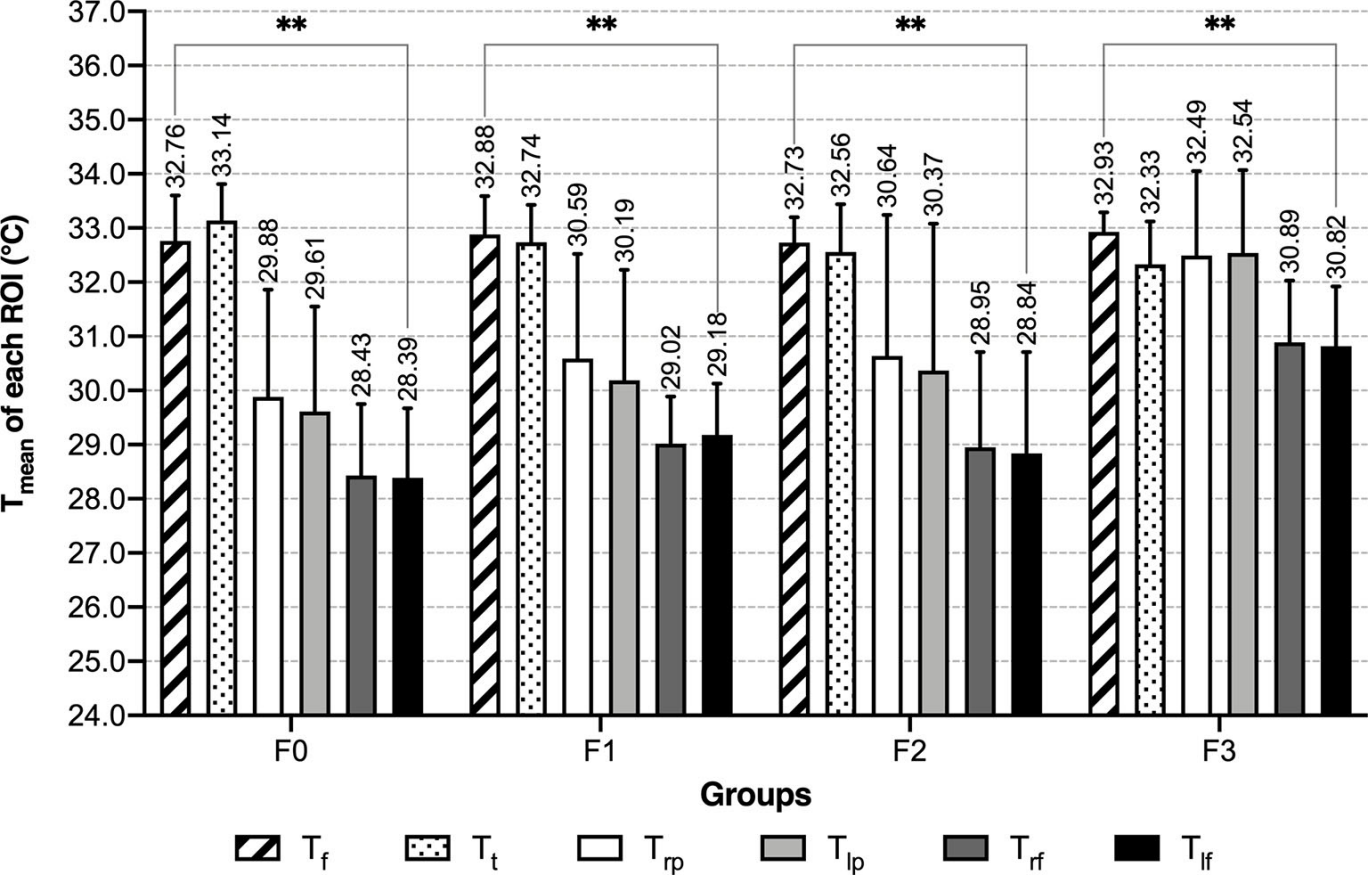
T_mean_ of each ROI of female groups - grouped by number of abnormal indexes. **: *P* < 0.01.

**Figure 7:**
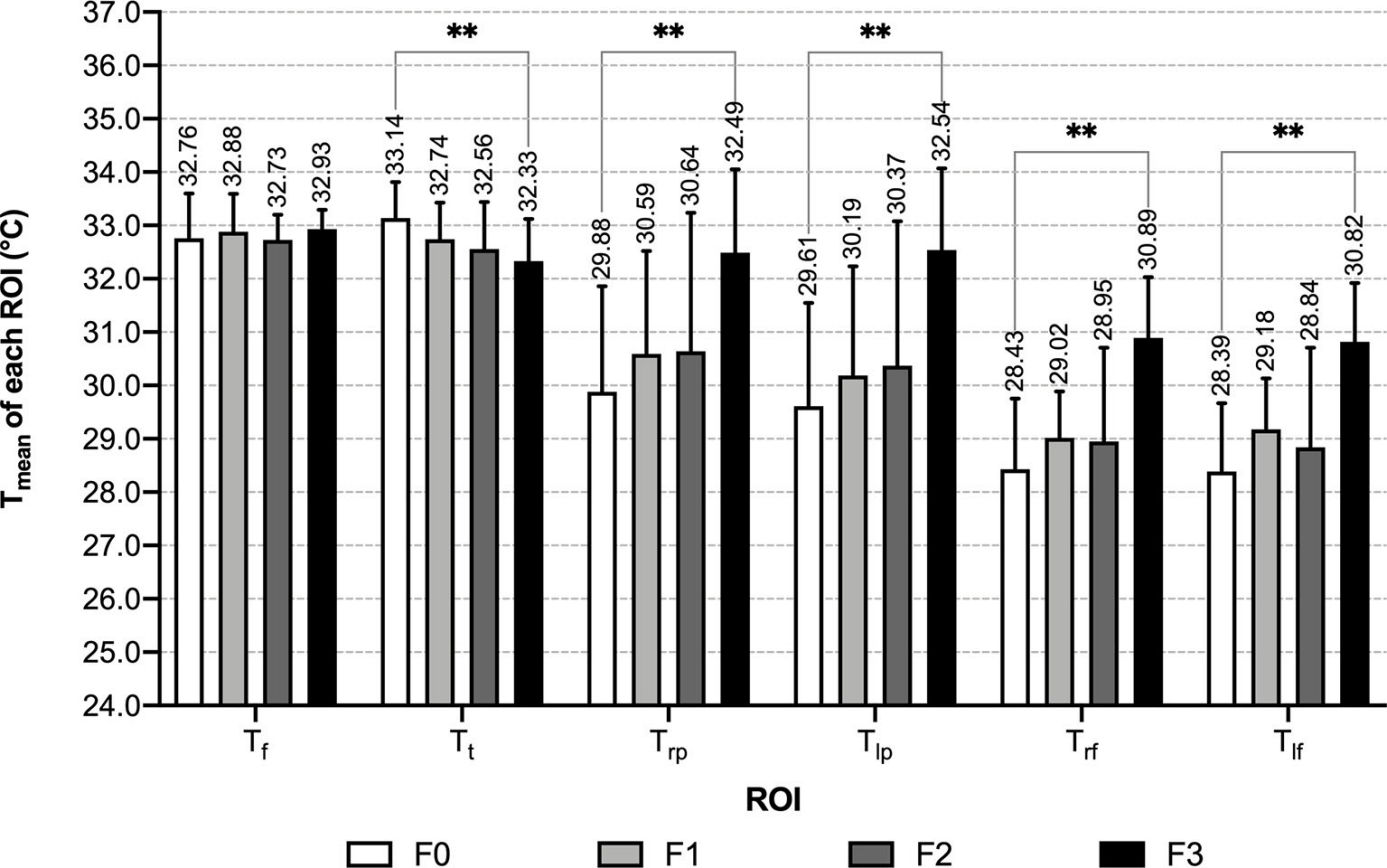
T_mean_ of each ROI of female groups - grouped by ROI. **: *P* < 0.01.

In female groups, characteristics of the temperature distribution of each ROI differ between groups with the different numbers of abnormal indexes. Along with the increase of abnormal metabolic indexes, the T_mean_ of bilateral palms and dorsum of feet increased, while the T_mean_ of anterior trunk decreased. In the normal group, the anterior trunk had the highest mean temperature, followed by the face, then palms and feet; in groups with 1 or 2 abnormal indexes, T_t_ became lower than T_f_; in the MS group, T_t_ continuously decreased, and T_mean_ of the palms increased to a level slightly higher than T_t_ (**Table 2** and **Figure 6**). T_f_ was the only ROI that showed no significant differences among the four female groups (**Table 2** and **Figure 7**). Compared with the normal female group, female MS patients had higher mean temperature in their bilateral palms and dorsum of feet, lower mean temperature in their anterior trunks (*P*<0.01) (**Supplementary Table 2**).

### 3.3 Correlation Analysis and Linear Regression Between Mean Temperature of ROI and Measurement Data & Laboratory Data

#### 3.3.1 Male Participants

For male participants, the results of correlation analysis showed positive correlations between T_f_ and DBP (r=0.256, *P*=0.014), T_rp_ & T_lp_ and BMI (with r-values of 0.239 and 0.234, respectively, *P<*0.05) and negative correlations between T_t_ and SBP, DBP, UA, TG, hip circumference, body mass, BMI and waist circumference (**Supplementary Table 3**). Unary linear regression was done between T_f_ & DBP, T_rp_ & BMI and T_lp_ & BMI (**Supplementary Table 4** and **Supplementary Figure 1**), details on the regression equations were listed in **Supplementary Data**.

#### 3.3.2 Female Participants

Correlation analysis on T_mean_ of each ROI and measurement & laboratory data of female participants are shown in **Table 3**. Multiple stepwise regression was carried out between T_mean_ and correlated indexes, details on the regression equations were listed in **Supplementary Data**.

**Table 3:** Pearson correlation analysis between T_mean_ of each ROI and measurement data & laboratory data in female groups.

	Pearson Correlation Coefficient (r)					
	T_f_	T_t_	T_rp_	T_lp_	T_rf_	T_lf_
Age (years)	-0.157	-0.392**	0.410**	0.421**	0.526**	0.481**
Height (cm)	0.229*	0.344**	0.014	-0.007	-0.013	0.018
Body mass (kg)	0.207*	-0.378**	0.340**	0.369**	0.319**	0.315**
BMI (kg/m2)	0.127	-0.514**	0.343**	0.381**	0.334**	0.317**
Waist circumference (cm)	0.069	-0.494**	0.308**	0.342**	0.297**	0.298**
Hip circumference (cm)	0.185	-0.346**	0.239*	0.253*	0.190	0.196
SBP (mmHg)	-0.001	-0.177	0.023	0.025	0.116	0.112
DBP (mmHg)	-0.071	-0.231*	0.043	0.049	0.081	0.071
FPG (mmol/L)	0.028	-0.219*	0.261*	0.274**	0.388**	0.384**
UA (μmol/L)	0.107	-0.224*	0.201	0.223*	0.236*	0.188
TC (mmol/L)	-0.124	-0.157	0.044	0.024	0.036	0.026
TG (mmol/L)	0.104	-0.374**	0.365**	0.377**	0.342**	0.356**
HDL-C (mmol/L)	-0.161	0.274**	-0.372**	-0.376**	-0.425**	-0.371**
LDL-C (mmol/L)	-0.101	-0.218*	0.145	0.132	0.187	0.141

Bold r-values mean that the correlations between the corresponding T_mean_ and measurement data and laboratory data are statistically significant. ^*^P < 0.05, ** P < 0.01.

## 4 Discussion

Our study demonstrated that: With the increasing number of abnormal metabolic indexes, the mean temperature gradually increased in the face, bilateral palms and dorsum of feet, and decreased in the anterior trunk. And for the MS patients, the temperature of the anterior trunk and bilateral palms became approximated. This specific tendency may indicate the severity of metabolic disorders.

The gender difference in temperature distribution was the basis of our subsequent analysis. In this study, there were obvious gender differences that the mean temperatures of face, palms and dorsum of feet were significantly higher in males than in females, while the mean temperature of anterior trunk showed the contrary characteristic. Several studies have also reported gender differences in temperature distribution. A study on healthy participants showed that the mean temperature of the chest was significantly higher in women than in men, while in the other analyzed body surface areas, including back, abdomen, and four limbs (anterior and posterior), the mean temperatures were significantly lower in women, which may be related to less skeletal muscle and thicker subcutaneous fat in females (20). Since we did not separate the anterior trunk region into chest and abdomen, the temperature characteristics of the anterior trunk of these two studies are not comparable, while the results that males have higher temperature in palms and dorsum of feet are consistent with this study. Another study found lower mean temperatures in the trunk and four limbs in the female group, which may have a bearing on higher body fat rates in females (21). The result of the lower mean temperature of the anterior trunk in females did not conform to our study. Consequently, further researches refer to the gender difference in body surface temperature distribution with a more detailed delimitation of ROI remain to be done.

The features of the thermal maps of the MS population were the key points of our study. And we did observe specific characteristic changes in the thermal maps of the MS population. There were few articles in allusion to the characteristic of infrared thermography of the MS population. Still, several studies on thermal characteristics of obesity, hyperlipidemia, hyperglycemia and hypertension have been done. Eduardo Borba Neves et al. (21) have discovered that, in males, the temperature in hands (anterior and posterior) had a tendency of increasing along with the body fat rate (BF%). Besides, BF% had a negative correlation with temperature in the anterior trunk, which was basically consistent with the results of our study. Eun-Mo Song et al. (22) found that the visceral fat area (VFA) was negatively correlated with the temperature of the acupoint CV4, which is located in the lower abdomen. This also corroborated the correlation between obesity and the decrease of temperature in the abdomen. Thiruvengadam J et al. (12) demonstrated that HDL was negatively correlated with the surface temperature of the left hand and anterior feet, which coincide with the conclusion of this study that severer metabolic disorder leads to higher mean temperature in palms and dorsum of feet. Aleck Ovechkin et al. (23) found the body surface temperature of the “Yin-Tang” acupuncture point which is located in the forehead negatively correlates with the severity of intracranial hypertension syndrome. This observation suggests varied temperature distribution characteristics of the face in hypertension patients compared with normal ones. While in this study, no change in the mean temperature of the face was found in MS participants, suggesting changes in body surface temperature of certain diseases might take place in some particular small areas, which could be covered when chosen ROIs are quite bigger. In summary, the characteristics of higher mean temperatures in palms and dorsum of feet, and lower mean temperature in the anterior trunk might be results of multiple pathological mechanisms as above. Studies on one particular metabolic disorder like hyperlipidemia, hypertension, and hyperglycemia may help in understanding the underlying mechanism of characteristic changes. However, clinical trials on large scales are still in lack, which conduces to a more accurate interpretation of the thermography characteristic of MS.

Through correlation analysis and linear regression, in both male and female groups, we found a positive correlation between the mean temperature of the palms and BMI, and negative correlations between mean temperature of the trunk and DBP, UA, TG, waist circumference, hip circumference, weight and BMI. These patterns can be consistent with the above-mentioned law of increasing palm temperature and decreasing trunk temperature with the aggravation of metabolic abnormalities. The unique results in male groups are: a positive correlation between T_f_ and DBP, and a negative correlation between T_t_ and SBP. In females, the unique results are: T_f_ was positively correlated with height and weight, the mean temperature of palms and dorsum of feet was positively correlated with hip circumference, UA, weight, BMI, waist circumference, FPG, TG and age, negatively correlated with HDL-C, and the average temperature of the trunk was negatively correlated with FPG, LDL-C, age and height. In general, the correlation between T_t_ and multiple metabolic indexes is obvious regardless of gender. While the mean temperature of palms and dorsum of feet showed correlations with a sizable amount of indexes only in the female group, and correlated with age at the same time. Given the uneven age baseline of female subjects, the relationship between palms and feet temperature and metabolic abnormalities still needs to be verified based on more homogeneous sample studies.

## 5 Limitations

It is also of note that heterogeneity was found among the age of four female groups. The average age of the female MS group was 55.58 ± 10.431 years old, mostly in perimenopausal and postmenopausal stages (start at 47.5 years old on average (24)). The particular age of this group may be due to the prevalence increase of MS in perimenopausal and postmenopausal women. A meta-analysis carried out by Hallajzadeh et al. (25) found the prevalence of MS among postmenopausal women was 37.17% and the pooled OR for MS in postmenopausal women in comparison with the premenopausal was 3.54. In perimenopausal and postmenopausal women, decreased estradiol level attenuates the protective effect of estradiol, which may account for the inclination of carbohydrate and lipid metabolism disorder and contribute to the development of MS (26, 27). However, due to the small sample size of this group, there remains the possibility that the sampling error led to the elder age of women in this group. Accordingly, further study with an enlarged sample size is necessary.

Owing to the retrospective nature of this study, the results could be influenced by time, temperature, humidity and state of equipment, etc. Due to the lack of research on MS using IRT, the sample size was not calculated in advance. While we calculated the sample size afterward based on the results of this study. Except for the comparison of T_lp_ between M0 and M3, T_t_ between F0 and F3, T_rf_ and T_lf_ between F2 and F3, the statistical powers of the other comparisons were higher than 0.80. Moreover, the sample size and age of each group were not evenly distributed, which may also have some bearing on the outcome. A methodological problem is that we did not capture the thermograph of the dorsum of feet in a vertical angle, for the retrospective nature, also. This could affect the output temperatures with camera (28), while since all of the thermographs were captured in approximate angles, the tendency of temperature changes we observed is still advisable.

## 6 Conclusion

This work was a pilot study of temperature characteristics in the MS population, and it proved the feasibility of screening and evaluating metabolic disorders through IRT. The key findings of this work were the gender difference in temperature distribution, the sequences of the mean temperature of the face, anterior trunk, palms and dorsum of feet in subjects of different metabolic conditions, the trends of the temperature changes in the above body parts with increasing number of abnormal metabolic indexes, and the certain differences in mean temperature of these body parts between the normal group and MS group. And the secondary findings are the correlations between measurement or laboratory data related to metabolic disorders and the mean temperature of different body parts. With the popularization of IRT and the deepening of the research, this correlation may help screen patients with metabolic disorders, further postponing the development of diseases.

## Data Availability Statement

The raw data supporting the conclusions of this article will be made available by the authors, without undue reservation.

## Ethics Statement

The studies involving human participants were reviewed and approved by Ethics Committee of Dongzhimen Hospital. The patients/participants provided their written informed consent to participate in this study.

## Author Contributions

All authors developed the concept for the study. Y-hX and W-zZ offered the design of this manuscript. RC provided suggestions. M-jG and Z-jC conducted the experiment. H-zX drafted the manuscript. B-yJ did literature search. B-hM provided technical support on the software in this study and guided the revision process of this manuscript. Y-cS reviewed the manuscript and made corrections. All authors contributed to the article and approved the submitted version.

## Funding

This work was supported by State Administration of Traditional Chinese Medicine JB063 programme and the Fundamental Research Funds for the Central Universities 2020-JYB-ZDGG-117 programme.

## Conflict of Interest

Author RC is employed by Beijing Wholelife Medical Science Co., Ltd.

The remaining authors declare that the research was conducted in the absence of any commercial or financial relationships that could be construed as a potential conflict of interest.

## Publisher’s Note

All claims expressed in this article are solely those of the authors and do not necessarily represent those of their affiliated organizations, or those of the publisher, the editors and the reviewers. Any product that may be evaluated in this article, or claim that may be made by its manufacturer, is not guaranteed or endorsed by the publisher.
