# Supplementary material for: A Preliminary Study on Infrared Thermograph of Metabolic Syndrome
Source: Front Endocrinol (Lausanne). 2022 Apr 12;13:851369. doi: 10.3389/fendo.2022.851369 (PMC9042650; doi:10.3389/fendo.2022.851369)
Supplement: Supplementary file 1 [file DataSheet_1.docx]

Supplementary Material

# Supplementary Data

For male groups, multiple stepwise regression was carried out between T_t_ and correlated indexes (body mass, BMI, waist circumference, hip circumference, SBD, DBP, UA and TG), the regression equation was established as below:

Ŷ=36.997-0.063X_1_-0.025X_2_-0.002X_3_

Where:

Ŷ represents T_t_ (℃);

X_1_ represents BMI (kg/m^2^);

X_2_ represents waist circumference (cm);

X_3_ represents UA (mmol/L).

It can be considered that BMI (X_1_), waist circumference (X_2_) and UA (X_3_) are the main factors that affect the temperature of the anterior trunk. R^2^ (coefficients of determination) =0.464. It is indicated that BMI, waist circumference and UA can explain the variation of T_t_ of this sample by 46.4%.

For female groups, multiple stepwise regression was carried out between T_mean_ and correlated indexes.

1. Regression equation between T_f_ and height & body mass:

Ŷ=27.78+0.031X.

Where:

Ŷ represents T_f_;

X represents height.

R^2^=0.052, P=0.028.

The height could explain 5.2% of the variation of T_f_.

1. Regression equation of T_rp_ and age, body mass, BMI, waist circumference, hip circumference, FPG, TG and HDL-C:

Ŷ=27.79+0.064X_1_-1.831X_2_+0.049X_3_.

Where:

Ŷ represents T_rp_ (℃);

X_1_ represents age (years);

X_2_ represents HDL-C (mmol/L);

X_3_ represents body mass(kg).

R^2^=0.322, P=0.000.

The age, HDL-C and body mass are the main factors that affect T_rp_. These factors above could explain 32.2% of the variation of T_rp_ of this sample.

1. Regression equation of T_lp_ and age, body mass, BMI, waist circumference, hip circumference, FPG, UA, TG and HDL-C:

Ŷ=26.891+0.064X_1_-1.826X_2_+0.058X_3_.

Where:

Ŷ represents T_lp_ (℃);

X_1_ represents age (years);

X_2_ represents HDL-C (mmol/L);

X_3_ represents body mass (kg).

R^2^=0.346, P=0.000.

The age, HDL-C and body mass are the main factors that affect T_lp_. These could explain 34.6% of the variation of T_lp_ of this sample.

1. Regression equation of T_rf_ and age, body mass, BMI, waist circumference, FPG, UA, TG and HDL-C:

Ŷ=29.059+0.069X_1_-1.606X_2_+0.058X_3_-0.05X_4_.

Where:

Ŷ represents T_rf_ (℃);

X_1_ represents age (years);

X_2_ represents HDL-C (mmol/L);

X_3_ represents body mass (kg);

X_4_ represents waist circumference (cm).

R^2^=0.474, P=0.000.

The age, HDL-C body mass and waist circumference are the main factors that affect T_rf._ And the factors above could explain 47.4% of the variation of T_rf_ of this sample.

1. Regression equation of T_lf_ and age, body mass, BMI, waist circumference, FPG, TG and HDL-C:

Ŷ=27.085+0.053X_1_-1.298X_2_+0.028X_3_.

Where:

Ŷ represents T_lf_ (℃);

X_1_ represents age (years);

X_2_ represents HDL-C (mmol/L);

X_3_ represents body mass (kg).

R^2^=0.371, P=0.000.

The age, HDL-C and body mass are the main factors that affect T_lf_ and could explain 37.1% of the variation of T_lf_ of this sample.

1. Regression equation of T_t_ and age, height, body mass, BMI, waist circumference, hip circumference, DBP, FPG, UA, TG and HDL-C:

Ŷ=30.727-0.095X_1_-0.015X_2_+0.030X_3_.

Where: Ŷ represents T_t_ (℃);

X_1_ represents BMI (kg/m^2^);

X_2_ represents age (years);

X_3_ represents height (cm).

R^2^=0.385, P=0.000.

This shows that BMI, age and height are the main factors that affect T_t_ and could explain 38.5% of the variation of T_t_ of this sample.

# Supplementary Tables

| **Supplementary Table 1** Comparison of T_mean_ of each ROI between different genders (℃; ‾x±s) | | | | | | | |
| --- | --- | --- | --- | --- | --- | --- | --- |
| Groups | n | T_f_ | T_t_ | T_rp_ | T_lp_ | T_rf_ | T_lf_ |
| Male groups (M0-M3) | 91 | 33.29±0.61** | 32.13±0.96** | 31.46±1.80** | 31.32±1.80** | 29.48±1.73* | 29.54±1.79* |
| Female groups (F0-F3) | 93 | 32.80±0.72 | 32.61±1.09 | 30.46±2.14 | 30.20±2.19 | 28.94±1.48 | 28.93±1.48 |

Note: Compared with female groups * *P*<0.05, ** *P*<0.01

| **Supplementary Table 2** Comparison of T_mean_ of each ROI between normal groups and MS groups (℃; ‾x±s) | | | | | | | |
| --- | --- | --- | --- | --- | --- | --- | --- |
| **Groups** | **n** | **T_f_** | **T_t_** | **T_rp_** | **T_lp_** | **T_rf_** | **T_lf_** |
| Normal male (M0) | 26 | 33.14±0.72 | 33.12±0.50 | 30.95±2.16 | 30.81±2.18 | 29.26±1.95 | 29.33±2.07 |
| MS male (M3) | 31 | 33.40±0.53 | 32.00±0.73** | 32.06±1.46 | 31.87±1.51^▲^ | 29.74±1.70 | 29.77±1.71 |
| *P* |  | 0.128 | 0.000** | 0.053 | 0.036^▲^ | 0.136 | 0.154 |
| Normal female (F0) | 50 | 32.76±0.84 | 33.14±0.67 | 29.88±1.98 | 29.61±1.94 | 28.43±1.32 | 28.39±1.28 |
| MS female (F3) | 12 | 32.93±0.36 | 32.33±0.79 | 32.49±1.56 | 32.54±1.53 | 30.89±1.14 | 30.82±1.10 |
| *P* |  | 0.282 | 0.004^▲▲^ | 0.000^▲▲^ | 0.000^▲▲^ | 0.000** | 0.000** |

^1^ Two-sample t-test was applied, α=0.05, ** *P*<0.01.

^2^ Wilcoxon rank- sum test was applied, α=0.05, ^▲^P<0.05.

| **Supplementary Table 3** Correlation analysis between T_t_ and measurement data & laboratory data in male group | | | | | | |
| --- | --- | --- | --- | --- | --- | --- |
| **Measurement data** | **Pearson Correlation Coefficient (r)** | ***P*** |  | **Laboratory data** | **Pearson Correlation Coefficient (r)** | ***P*** |
| Age (years) | -0.106 | 0.318 |  | FPG (mmol/L） | -0.093 | 0.381 |
| Height (cm) | 0.006 | 0.956 |  | UA (μmol/L） | **-0.310**** | 0.003 |
| Body mass (kg) | **-0.606**** | 0.000 |  | TC (mmol/L） | -0.08 | 0.453 |
| BMI (kg/m^2^) | **-0.650**** | 0.000 |  | TG (mmol/L） | **-0.366**** | 0.000 |
| Waist circumference (cm) | **-0.647**** | 0.000 |  | HDL-C (mmol/L） | 0.184 | 0.082 |
| Hip circumference (cm) | **-0.538**** | 0.000 |  | LDL-C (mmol/L） | -0.046 | 0.67 |
| SBP (mmHg) | **-0.352**** | 0.001 |  |  |  |  |
| DBP (mmHg) | **-0.320**** | 0.002 |  |  |  |  |

** *P*<0.01

| **Supplementary Table 4** Unary linear regression of T_f,_ T_rp,_ T_lp_ with correlated measurement data & laboratory data | | |
| --- | --- | --- |
| T_f_ with DBP |  | *P*=0.014 |
| R^2^=0.066 |  |  |
| T_rp_ with BMI |  | *P*=0.023 |
| R^2^=0.057 |  |  |
| T_lp_ with BMI |  | *P*=0.025 |
| R^2^=0.055 |  |  |

Note: R^2^ represents the coefficient of determination

# Supplementary Figure


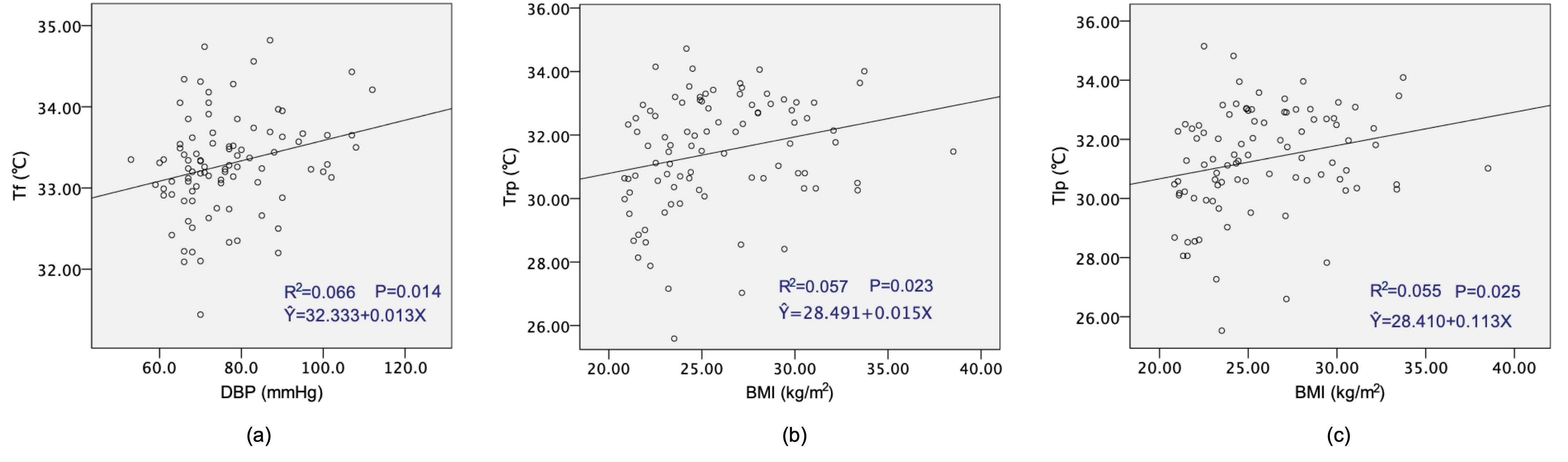


**Supplementary Figure 1** (a) Unary linear regression analysis and scatter plot of T_f_ to DBP of male groups. (b) Unary linear regression analysis and scatter plot of T_rp_ to BMI of male groups. (c) Unary linear regression analysis and scatter plot of T_lp_ to BMI of male groups
